# Extracellular vesicles in gastric cancer: role of exosomal lncRNA and microRNA as diagnostic and therapeutic targets

**DOI:** 10.3389/fphys.2023.1158839

**Published:** 2023-08-16

**Authors:** Chengyao Jiang, Jianjun Zhang, Wentao Wang, Zexing Shan, Fan Sun, Yuen Tan, Yilin Tong, Yue Qiu

**Affiliations:** ^1^ Department of Gastric Surgery, Liaoning Cancer Hospital and Institute, Cancer Hospital of China Medical University, Shenyang, China; ^2^ Medical Oncology Department of Gastrointestinal Cancer, Cancer Hospital of China Medical University, Liaoning Cancer Hospital and Institute, Shenyang, Liaoning, China

**Keywords:** extracellular vesicles, nanoparticles, gastric cancer, biomarker, circRNAs

## Abstract

Extracellular vesicles (EVs), including exosomes, play a crucial role in intercellular communication and have emerged as important mediators in the development and progression of gastric cancer. This review discusses the current understanding of the role of EVs, particularly exosomal lncRNA and microRNA, in gastric cancer and their potential as diagnostic and therapeutic targets. Exosomes are small membrane-bound particles secreted by both cancer cells and stromal cells within the tumor microenvironment. They contain various ncRNA and biomolecules, which can be transferred to recipient cells to promote tumor growth and metastasis. In this review, we highlighted the importance of exosomal lncRNA and microRNA in gastric cancer. Exosomal lncRNAs have been shown to regulate gene expression by interacting with transcription factors or chromatin-modifying enzymes, which regulate gene expression by binding to target mRNAs. We also discuss the potential use of exosomal lncRNAs and microRNAs as diagnostic biomarkers for gastric cancer. Exosomes can be isolated from various bodily fluids, including blood, urine, and saliva. They contain specific molecules that reflect the molecular characteristics of the tumor, making them promising candidates for non-invasive diagnostic tests. Finally, the potential of targeting exosomal lncRNAs and microRNAs as a therapeutic strategy for gastric cancer were reviewed as wee. Inhibition of specific molecules within exosomes has been shown to suppress tumor growth and metastasis in preclinical models. In conclusion, this review article provides an overview of the current understanding of the role of exosomal lncRNA and microRNA in gastric cancer. We suggest that further research into these molecules could lead to new diagnostic tools and therapeutic strategies for this deadly disease.

## 1 Introduction

Gastric cancer (GC) represents a prevalent malignancy of the gastrointestinal tract with increased morbidity and mortality rates each year (Wu H. et al., 2021; Yeoh and Tan, 2022). Based on Lauren’s histological classification, GC is divided into two subtypes: intestinal and diffuse gastric adenocarcinoma (Fujiyoshi et al., 2021; Zhu et al., 2021). The GC progression usually passes through chronic non-atrophic gastritis, atrophic gastritis, intestinal epithelial hyperplasia, atypical hyperplasia, and ultimately invades GC (Wang et al., 2021a; Choi et al., 2022). *H. Pylori* infection stands as the primary risk factor for GC (Li M. et al., 2022; Bessède and Mégraud, 2022; Li Z. et al., 2022; Zhu et al., 2022). Additionally, diet-related risk factors like high salt diet, reduced vegetable intake and alcohol abuse impact the occurrence and prognostic outcome of GC (Bouras et al., 2022; Li et al., 2022c; Liatsos et al., 2022; Pappas-Gogos et al., 2022). The process of GC development, progression, invasion, and metastasis is also closely linked to changes in gene expression, gene mutations, and epigenetic alterations (Herrera-Pariente et al., 2021; Liu Y. et al., 2022; Jelski and Mroczko, 2022). Despite GC being preventable, the prevalence of early screening is still low, leading to a high percentage of GC patients being in the late-stage tumor by initial diagnosis, which hinders surgical intervention success, exhibits an increased metastasis rate and carries a dismal prognosis (Li G. Z. et al., 2022; Ferreira et al., 2022). The primary treatment strategies for early to mid-stage GC are surgery, adjuvant chemotherapy, and radiotherapy (Puliga et al., 2021; Fong et al., 2022). Although these treatments achieved longer recurrence and metastasis time, approximately 50% of patients relapse and eventually die within 2 years following surgery (Li K. et al., 2021; Seeneevassen et al., 2021). The 5-year survival rate for gastric cancer depends on the stage of the cancer at the time of diagnosis. According to the American Cancer Society’s estimates for 2021 (Sung et al., 2021), the overall 5-year survival rate for gastric cancer is about 32%. However, this survival rate varies widely depending on the stage of the cancer: Localized gastric cancer: The 5-year survival rate is about 68%. Regional gastric cancer: The 5-year survival rate is about 31%. Distant gastric cancer: The 5-year survival rate is about 5%. Hence, it is crucial to investigate molecular diagnostic indicators, prognostic analysis factors, and individualized treatment markers for GC. Gastric cancer has different stages according to the spread of cancer cells. The stage is critical in determining treatment and prognosis. The stages of gastric cancer are early, mid and late stage. Early-stage gastric cancer has cancer limited to the inner stomach layers, without invasion into other organs and mild symptoms such as nausea or indigestion. Mid-stage gastric cancer impacts deeper layers of the stomach lining, causing abdominal pain, weight loss, bloating and fatigue. Late-stage gastric cancer has spread to other organs, causing severe symptoms such as vomiting blood, difficulty swallowing and jaundice. Treatment strategies vary depending on the stage and location of cancer. Treatment could include endoscopic resection, gastrectomy, chemotherapy or radiation therapy. Recent advancements in treatment have enhanced the treatment options for gastric cancer (Cai et al., 2022).

Exosomes are a form of secretory vesicles enriched with a vast amount of proteins, lipids, nucleic acids, and other biological information material that ranges from 30 to 150 nm in diameter and can facilitate the transfer of intercellular information signals (Dad et al., 2021; Wei et al., 2021). Almost all cells secrete exosomes, and they are present in nearly all body fluids (Yu W. et al., 2021; Lin et al., 2021; Mohammadi et al., 2021). Exosomes play an essential role in different biological processes, serving as a promoter of the transfer of information between cells; more importantly, exosomes facilitate the transmission of information among cancer cells that remain noteworthy in the TME (Noonin and Thongboonkerd, 2021; Ansari et al., 2022). In the TME, exosomes assume a vital function in intercellular information transfer, which enables the transmission of information between cancer cells, carries molecular signals into the recipient cells, promotes various biological processes such as tumor growth, metastasis, invasion, angiogenesis, tumor innervation, and chemotherapeutic drug resistance (Yang G. H. et al., 2021; Pathania et al., 2021; Sun et al., 2021; Zhao et al., 2021; Huang et al., 2022).

Circular RNAs (circRNAs) comprise a kind of endogenous ncRNAs that emerged in the 1970s and were initially believed to result from RNA variable splicing errors (Ebbesen et al., 2017; Tsitsipatis and Gorospe, 2021). However, with advances in high-throughput sequencing, genomic micro-array and bioinformatics, CircRNAs were found to be widely present in eukaryotes and involved in numerous diseases like neurological, cardiovascular disease, and cancers (Song et al., 2020; Zhang C. et al., 2021; Cheng et al., 2021; Tang et al., 2021). Moreover, it plays an important role in several pathophysiological functions such as aging of living organisms and neurodevelopment (Meng et al., 2020; Kim et al., 2021). Long non-coding RNAs (lncRNAs) are a kind of ncRNAs with superior length exceeding 200 nucleotides (Ma X. et al., 2022; Wang et al., 2022a). These lncRNAs are prevalent within human genes, and though they cannot encode proteins, they participate in forming complex regulatory networks of gene expression and regulate various biological processes (Kong et al., 2022; Taghehchian et al., 2022). It was discovered that circRNAs and lncRNAs had crucial biological impacts in the GC development (Ma Q. et al., 2022; Wang et al., 2022b; Wang X. et al., 2022; Hu et al., 2022; Zhou et al., 2022). It has been demonstrated that exosomal microRNAs (miRNAs) serve a crucial function in the initiation of GC (Mirzakhani et al., 2019; Wu et al., 2019; Lin et al., 2020; Yang et al., 2020), as well as in the early diagnosis and prognostic assessment of GC (Li B. et al., 2021; Dou et al., 2021; Guo et al., 2021; Yang et al., 2022). Recent studies have shown that exosomal circRNAs and lncRNAs are involved in the GC tumorigenesis and malignant progression and the diagnosis and prognosis of GC. In this review, we provide an overview and highlight the progress of research related to the role of exosomal circRNAs and lncRNAs in the development, diagnosis, prognosis assessment, and treatment of GC.

## 2 Overview of exosomes

### 2.1 Structure and formation of exosomes

Extracellular vesicles (EVs) have been reported to be membranous structures released into the extracellular environment by all studied organisms and cell types (Verweij et al., 2021). The EV family is diverse and has been categorized based on subcellular origin, size, and composition (Verweij et al., 2019). The EV subtypes include endosomal-derived vesicles such as the polyvesicular endosomal-derived exosomes (50–150 nm in diameter) and secretory autophage-derived EVs, exosomes and other microbubbles that sprout from the plasma membrane (PM), intermediate residues released by dividing cells, transitional bodies released by transitional cells, apoptotic bodies, and large tumor bodies (Zhang et al., 2020a; Popowski et al., 2020). Microvesicles, which are shed from the plasma membrane of different cell types, measure approximately 100–1,000 nm in diameter (Mirzakhani et al., 2019; Yang et al., 2020). Apoptotic vesicles, with diameters ranging from 1,000 to 5,000 nm, are released by apoptotic cells (Mirzakhani et al., 2019; Yang et al., 2020). Exosomes, which have an endosomal origin and sizes ranging from ∼40 to 160 nm in diameter (∼100 nm on average), are released by cells into the extracellular environment. These are characterized by their typical cup-shaped morphology and encapsulated by a lipid bilayer (Popowski et al., 2020). Exosomes contain biological information such as nucleic acids (miRNA, mRNA, *etc.*), proteins, and lipids, and are highly heterogeneous (Wu et al., 2019; Lin et al., 2020). Production of exosomes involves a unique intracellular regulatory process, and once secreted into the extracellular space, their composition and function can be determined.

**FIGURE 1:**
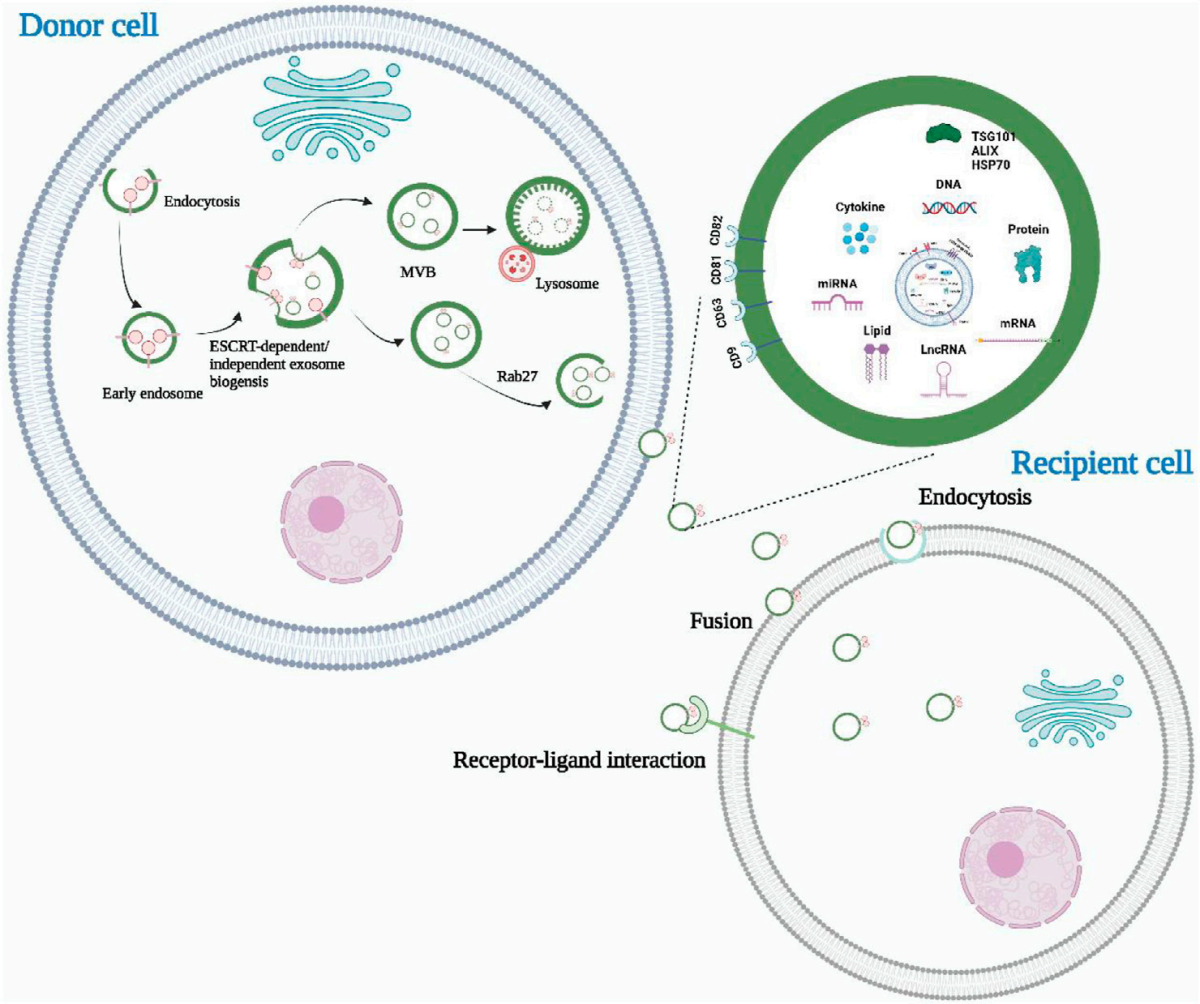
The biogenesis, release, and content of exosomes. Exosomes are derived from intracellular multivesicular bodies, which are fused to the plasma membrane and then partly degraded by lysosomes and partly released outside the cell to become exosomes. There are three main ways of information transfer between exosomes and target cells. Exosomal membrane proteins interact with target cell membrane proteins to activate intracellular signaling pathways, fuse directly with the target cell membrane, or transmit the genetic information carried directly to the recipient cell. Almost all cell types can secrete exosomes, which also contain a wide range of nucleic acids, proteins and lipids. Their surface markers mainly include CD63, CD81, CD9, Alix, TSG101, and HSP70.

The process of exosome formation is complex and involves several stages (Figure 1). After endocytosis, the endocytic cell membrane forms several vesicles that subsequently fuse to form the early endosome. The early endosomal membranes depress and bud inward, forming the late endosome and its intraluminal vesicles (ILVs) (Ranjbaran et al., 2019). Multivesicular bodies (MVBs) are ILVs-rich endosomes that have two destinations. A proportion of MVBs fuse with lysosomes and their content undergoes degradation (Phan et al., 2018; Meng et al., 2019). The other portion fuses with the cell membrane and releases ILVs into the extracellular environment, which are known as exosomes (Wan et al., 2018; Logozzi et al., 2019).

There are currently two main exosome formation pathways that are based on ILVs. The first is the endosomal sorting complex required for transport (ESCRT) formation pathway (Li et al., 2022e; Li and Ma, 2022; Wu et al., 2022). The ESCRT complexes help to recognize, sort, and transport ubiquitinated membrane proteins into the endosome. ESCRT consists of approximately 20 proteins that bind to auxiliary proteins to form four complexes (ESCRT-0, ESCRT-Ⅰ, ESCRT-Ⅱ, and ESCRT-Ⅲ) (Song et al., 2022a; Yu H. et al., 2022). ESCRT-0, which is composed of the hepatocyte growth regulator tyrosine kinase substrate (HRs) and signal transduction bridging molecule (STAM), recognizes ubiquitinated proteins in the endosomal membrane and interacts with lattice proteins to enrich them. ESCRT-Ⅰ and ESCRT-Ⅱ induce endosomal membrane loading, while ESCRT-Ⅲ shears the bud neck to induce vesicle separation. The auxiliary protein VPS4 plays a role in the ESCRT isolation and reassociation cycle (Kimiz-Gebologlu and Oncel, 2022). The second pathway is an ESCRT-independent pathway that assists in generating MVBs and ILVs through, for example, ceramide (Channon et al., 2022; Srivastava et al., 2022). Neutral sphingomyelinase (NSMASE) mediates the hydrolysis of sphingomyelin to produce ceramide. Inhibition of neutral sphingomyelinase decreases ceramide production, which in turn decreases the inward budding of MVBs membranes, ultimately leading to decreased exosome release. This suggests that ceramide forms exosomes in an ESCRT-independent manner (Yu D. et al., 2022).

### 2.2 Biological functions of exosomes

Exosomes are small extracellular vesicles that facilitate intercellular communication by transporting biomolecules involved in physiological or pathological processes from the parent cell to the recipient cell. Current research has identified three main modes of interaction between exosomes and recipient cells. Firstly, exosomal membrane proteins can bind to specific receptors on the surface of target cells and activate intracellular signaling pathways (Soltész et al., 2021; He et al., 2022). Secondly, exosomes can fuse directly with target cell membranes, releasing contents such as proteins, mRNAs, and miRNAs from the parent cell into the recipient target cell (Li C. et al., 2021; Thakur et al., 2022). Lastly, recipient cells can internalize exosomes via endocytosis, thereby regulating their basic functions and gene expression (Chen et al., 2021a; Attaran and Bissell, 2022). The role of exosomes is highly dependent on the content of their cargo, which includes various proteins of maternal cell origin. As such, exosomal proteins have been isolated, detected, and characterized to reflect the physiological and pathological status of maternal cells. Consequently, exosomal proteins have become non-invasive diagnostic and prognostic biomarkers for many types of diseases (Liu et al., 2021; Mao et al., 2021). Additionally, due to their structural and compositional similarity to cell membranes, good biocompatibility, and ability to penetrate deep into tissues while evading the immune system, exosomes have emerged as ideal drug nanocarriers. Compared to drug carriers such as liposomes, exosomes have low toxicity and can deliver drugs to target organs more effectively (Chen et al., 2021b; Zhang H. et al., 2021; Isaac et al., 2021). Liposomes have a tendency to accumulate in the liver and spleen, leading to off-target effects and reduced drug efficacy (Zhang et al., 2019). Additionally, liposomes encounter clearance by the immune system, resulting in a shorter half-life in circulation compared to exosomes (Yim et al., 2021). Immune responses induced by liposomes impose limitations on their repeated dosing for long-term treatments (Yim et al., 2021).

## 3 Overview of circRNAs

### 3.1 Molecular types and characteristics of circRNA

**FIGURE 2:**
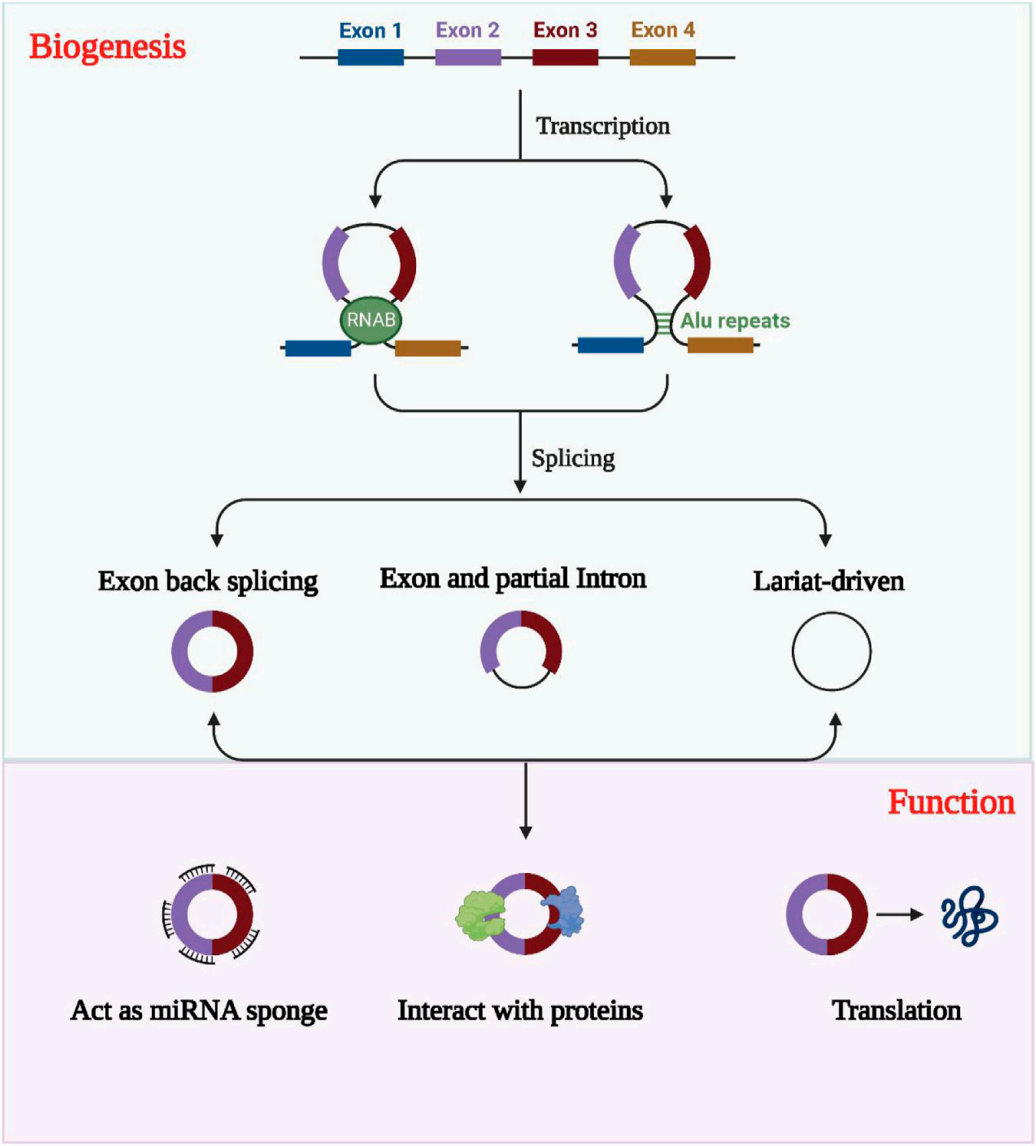
The formation, classification and biological functions of exosomal circRNAs. CircRNAs are usually classified into EIciRNAs, EcircRNAs and ciRNAs according to their constituents, which are derived from exons and introns, respectively, and all three are present in pre-mRNA. circRNAs can act as miRNA sponges to regulate the expression of downstream genes. Exosomal circRNAs were transported into the target cells and bind to RNA-binding proteins and regulate parental gene expression. CircRNAs with open reading frames also have the ability to encode polypeptides.

Compared to linear RNAs, circular RNAs (circRNAs) have a distinct structure in which the 5′ and 3′ends are directly covalently joined to form a loop structure devoid of a 3′ tail or 5′cap structure (Qu et al., 2015; Salzman, 2016). These non-coding RNAs are often derived from one or more exons and are relatively stable due to their resistance to nucleic acid exonucleases compared to linear RNAs, displaying greater sequence conservation (Qu et al., 2017; Zhao et al., 2022). Although circRNAs display variable expression levels, their tissue, cell, and temporal specificity reveals they play a crucial regulatory role in transcription and post-transcription processes, competing with endogenous RNAs (ceRNAs) to regulate gene expression (Tang and Hann, 2020; Wen et al., 2020; Shao et al., 2021). The formation of circRNAs involves three categories: circRNAs formed by intronic sequences (ciRNA), circRNAs formed by exonic sequences (ecircRNA), and circRNAs formed by both intronic and exonic sequences (elciRNA), as illustrated in Figure 2 (Lyu and Huang, 2017; Chen et al., 2022; Li W. et al., 2022).

### 3.2 Function of circRNA

CircRNA functions as a microRNA molecular sponge as it binds complementarily to miRNAs, and this is among its most typical functions (Lyu and Huang, 2017; Saikishore et al., 2020). Through this binding, circRNAs inhibit miRNAs’ biological activities. CircRNAs can also interact indirectly or directly with proteins, contributing to the development of diseases via a range of mechanisms. For instance, they can act as protein inducers or antagonists to regulate protein functions (Zhou W. Y. et al., 2020; Huang et al., 2020). Additionally, circRNAs can translate proteins, contrary to previous beliefs. Despite being thought to lack a suitable internal ribosome entry site and a 3′ tail or a 5′ cap structure, circRNAs have a covalent closed loop structure, making them capable of translating proteins. Recent research has demonstrated that several circRNAs can translate proteins. (Prats et al., 2020; Sinha et al., 2021; Liu X. et al., 2022).

## 4 Overview of lncRNAs

### 4.1 Classification of lncRNAs

Studies have demonstrated that approximately 70% of the human genome sequence is transcribed, where less than 2% has protein-coding functionality, while the remaining 98% comprises non-coding RNAs which lack coding capacity (Sun and Feinberg, 2021; Mercer et al., 2022). Non-coding RNAs (ncRNAs) can be categorized fundamentally into two sets: caretaker ncRNAs and regulatory ncRNAs (Born et al., 2020; Jantrapirom et al., 2021). Caretaker ncRNAs are typified as stably expressed, functioning significantly to sustain cellular survival, including transfer RNA, ribosomal RNA, and small nucleolar RNA (Wang et al., 2021b; Onoguchi-Mizutani and Akimitsu, 2022). In contrast, regulatory ncRNAs comprise mainly of miRNAs and long non-coding RNAs (lncRNAs), where lncRNAs account for 80% of ncRNAs and perform diverse functions at different levels of gene expression, including epigenetic, transcriptional and post-transcriptional regulation (Guo et al., 2020; Wu M. et al., 2021; DeSouza et al., 2021). The lncRNAs are highly numerous and exhibit diversity in terms of modes of actions. Currently, there is no standardized classification for lncRNAs, and classification relies primarily on molecular size, localization and function. LncRNAs may be classified based on their cellular localization, namely, cytosolic lncRNAs and cytoplasmic lncRNAs. The roles and regulations of lncRNAs differ with their cellular localization (Zhang et al., 2020b; Lu Q. et al., 2021). Based on the location of lncRNAs in the genome concerning protein-coding genes, they can be categorized into righteous lncRNAs, antisense lncRNAs, bidirectional lncRNAs, intragenic lncRNAs, and intergenic lncRNAs (MacDonald and Mann, 2020; Sun and Ye, 2020). Finally, lncRNAs may be classified as decoy molecules, guide molecules, and backbone molecules according to their molecular functions. Decoy molecules reduce the transcription of downstream genes by inducing transcription factors to bind to them (Qu et al., 2020). Guide molecules bind with molecules on the same site, directing them to the site of action, thereby enhancing the transcriptional activity of downstream genes.

### 4.2 Regulatory functions of lncRNAs

Long non-coding RNAs (lncRNAs) are ubiquitously present in various organisms. Unlike messenger RNAs (mRNAs), lncRNAs are expressed at relatively low levels and exhibit minimal sequence conservation. Initially, lncRNAs were considered as transcriptional “noise” with no discernible function in the genome. However, subsequent research has demonstrated their specific spatiotemporal expression patterns, which are restricted to particular stages of growth and development, tissues, and cell types, suggesting that lncRNAs play an essential role in cell differentiation and growth of multicellular organisms (Guo et al., 2019; Ramnarine et al., 2019). The molecular structure of lncRNAs determines their function, and their sequence specificity and complex structure enable them to interact with DNA, RNA, and protein molecules, influencing gene expression at almost all stages of gene expression (Kong et al., 2019; Lozano-Vidal et al., 2019). They are capable of participating in multiple levels of regulation, including epigenetic modifications, transcriptional and post-transcriptional levels of gene regulation.

**FIGURE 3:**
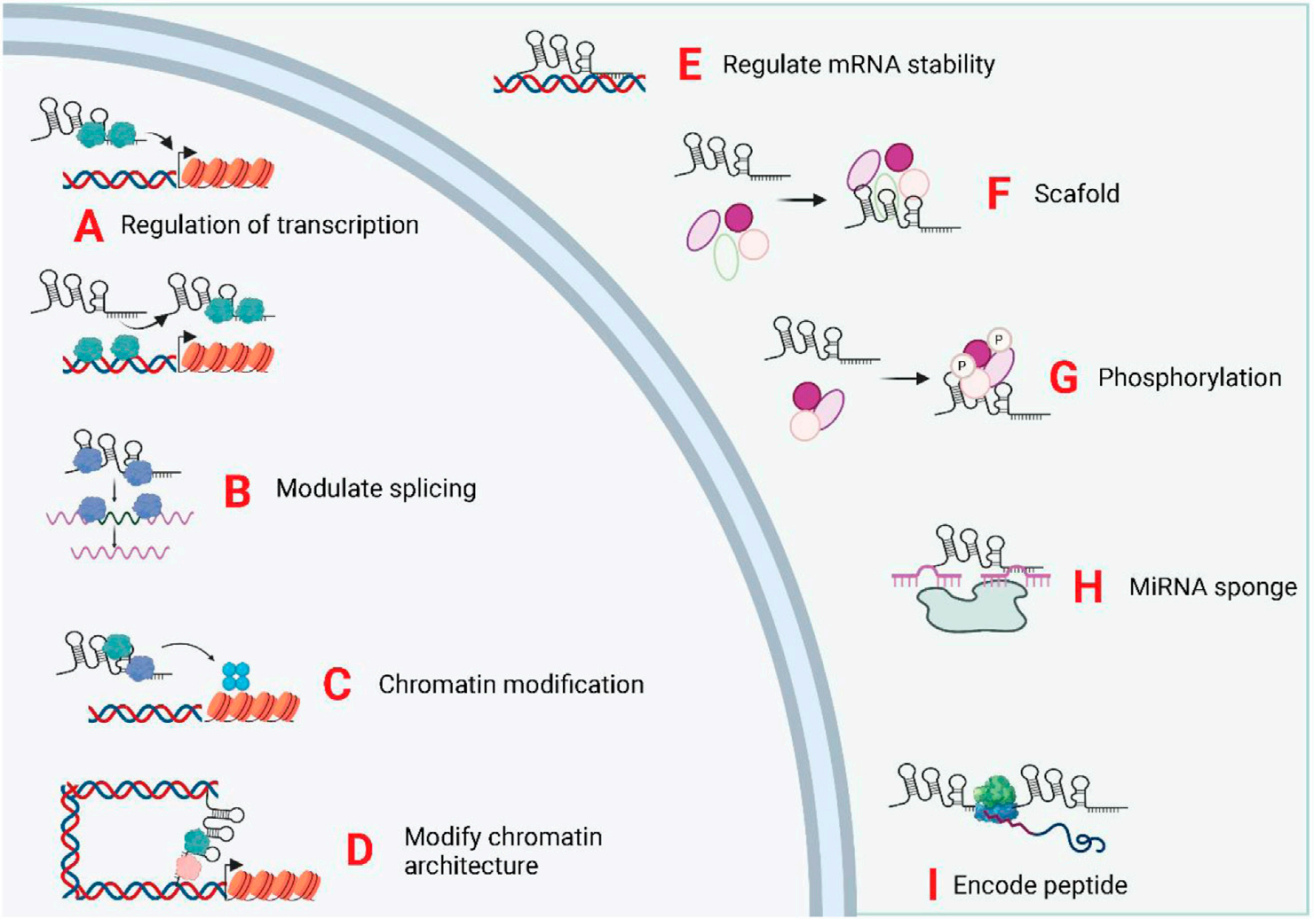
Biological functions of exosomal LncRNAs. The subcellular localization of LncRNA determines its biological functions. The exosomal, lncRNA were trasnported into the target cells and induce or repress gene transcription by directing transcription factors **(A)**, controlling splicing of pre-mRNAs **(B)**, mediating chromatin/histone modifications **(C)**, and modifying chromatin structure **(D)**. In the cytoplasm, lncRNAs can regulate mRNA stability **(E)**, act as scaffolds for ribonucleoprotein complexes **(F)**, mediate protein phosphorylation **(G)**, act as miRNA sponges **(H)**, and encode micropeptides **(I)**.

Epigenetic inheritance refers to the lack of changes in genomic DNA sequence, but heritable changes in the single gene expression, mainly comprising DNA methylation, histone modifications, and genomic imprinting (Li M. et al., 2019; Gugnoni and Ciarrocchi, 2019). Long non-coding RNA molecules can bind to DNA or protein complexes associated with chromatin modifications to neighboring genes and subsequently modulate their expression via acting in cis or trans (Li J. et al., 2019). Transcriptional regulation by lncRNAs constitutes a vital component of eukaryotic gene expression regulation and is the most prevalent mode of gene expression regulation (He et al., 2018; Li et al., 2018; Yi et al., 2019). LncRNAs regulate transcriptional processes via several different mechanisms. LncRNAs can act as miRNA sponges, and antisense lncRNAs form a protective mechanism by binding to the 3′UTR site of mRNAs. LncRNAs can also complementarily bind to mRNAs, mediating their degradation. LncRNAs act as miRNA sponges and protein sponges. The lncRNAs that serve as miRNA sponges are denoted as ceRNAs. CeRNAs modulate downstream gene expression positively via competitively binding miRNAs and reducing the effect of miRNAs on target mRNAs (Wang L. et al., 2019; Zhang et al., 2019a; Xiong et al., 2019). Similarly, lncRNAs that act as protein sponges inversely regulate the expression level of target genes by competitively binding to target mRNAs with a certain group of proteins (Wang, 2018; Lorenzi et al., 2019). Recently, some lncRNAs with open reading frames have been shown to encode polypeptides (Ye et al., 2020). The biological functions of lncRNAs was displayed in Figure 3.

### 4.3 Epigenetic marks associated with microRNAs and lncRNAs

Emerging evidence suggests that epigenetic modifications play a crucial role in the diagnostic and therapeutic potential of exosomal long non-coding RNA (lncRNA) and microRNA in gastric cancer. Epigenetic marks, such as DNA methylation, histone modifications, and chromatin remodeling, have been shown to regulate the expression and function of lncRNAs and microRNAs implicated in gastric cancer. These modifications can lead to aberrant gene expression patterns and contribute to the initiation and progression of gastric cancer.

DNA methylation, one of the most extensively studied epigenetic modifications, has been linked to the silencing of tumor suppressor lncRNAs and microRNAs in gastric cancer. Hypermethylation of promoter regions can inhibit the expression of these molecules, promoting tumor growth and metastasis (Esteller, 2007). On the other hand, hypomethylation of certain genomic regions may induce the overexpression of oncogenic lncRNAs and microRNAs, affecting key cellular processes in gastric cancer (Stram and Payne, 2016).

Histone modifications, including acetylation, methylation, and phosphorylation, have also been implicated in the regulation of exosomal lncRNAs and microRNAs in gastric cancer. For example, histone deacetylases (HDACs), which remove acetyl groups from histones, can repress the expression of tumor suppressor lncRNAs and microRNAs in gastric cancer cells (Zhao et al., 2023). Conversely, histone methyltransferases and demethylases have been found to modulate the expression of oncogenic lncRNAs and microRNAs, influencing cancer progression (Yi, 2022).

Furthermore, chromatin remodeling factors, such as DNA methyltransferases and histone modifiers, can impact the packaging and accessibility of lncRNA and microRNA genes, thereby influencing their expression profiles in gastric cancer cells (Carter et al., 2016). Alterations in chromatin structure resulting from these remodeling processes can affect the release and uptake of exosomes containing specific lncRNAs and microRNAs, potentially influencing intercellular communication within the tumor microenvironment. Considering the diagnostic potential, the epigenetic modifications of exosomal lncRNAs and microRNAs hold promise as non-invasive biomarkers for gastric cancer. The detection of specific DNA methylation patterns or histone modification signatures in exosomes could provide valuable information for early diagnosis, prognosis, and prediction of response to therapy (Qu et al., 2013).

Therapeutically, targeting the epigenetic marks associated with exosomal lncRNAs and microRNAs represents a promising strategy for gastric cancer treatment. Epigenetic modulators, such as DNA methyltransferase inhibitors or histone deacetylase inhibitors, can effectively reverse aberrant epigenetic patterns and restore the expression of tumor suppressor lncRNAs and microRNAs. This approach may help overcome resistance to conventional therapies and enhance therapeutic response in gastric cancer patients. In conclusion, the epigenetic aspects related to exosomal lncRNA and microRNA in gastric cancer provide important insights into the diagnostic and therapeutic potential of these molecules. Understanding the influence of epigenetic modifications on the expression and function of these exosomal components may pave the way for the development of personalized diagnostic tools and novel targeted therapies for gastric cancer.

### 4.4 Antagonistic interactions between lncRNAs and microRNAs

Antagonistic interactions between long non-coding RNAs (lncRNAs) and microRNAs (miRNAs) represent a complex regulatory network that plays a crucial role in gene expression and cellular processes. While miRNAs typically function as negative regulators of gene expression by binding to target messenger RNA (mRNA) molecules and inhibiting their translation or promoting their degradation, lncRNAs can have diverse functions, including acting as molecular sponges or decoys for miRNAs.

One mode of antagonistic interaction between lncRNAs and miRNAs involves the competitive binding of lncRNAs to miRNAs. Through the presence of miRNA response elements (MREs), lncRNAs can act as molecular sponges, sequestering miRNAs and preventing them from binding to their target mRNAs. This results in the de-repression of miRNA target genes and the subsequent upregulation of gene expression (Salmena et al., 2011). This antagonistic relationship allows lncRNAs to fine-tune the activity of miRNAs and impact gene regulatory networks. Another mode of interaction involves the direct base pairing between lncRNAs and miRNAs, leading to the inhibition of miRNA activity. In this case, lncRNAs can act as competitive inhibitors, binding to miRNAs and preventing them from associating with their target mRNAs. This interference disrupts the RNA-induced silencing complex (RISC) machinery and hampers the gene silencing capabilities of miRNAs (Cesana et al., 2011). This antagonistic interplay expands the regulatory repertoire of lncRNAs in modulating gene expression and cellular processes. Furthermore, some lncRNAs have been found to act as miRNA-directed guides, directing miRNAs to specific target sites on mRNA molecules. This interaction allows lncRNAs to function as “miRNA sponges with a target” or “let-7 sponges.” The presence of complementary sequences between lncRNAs and miRNAs facilitates the recruitment of miRNAs to specific mRNA targets, influencing their stability and translation efficiency (Karreth et al., 2011). This antagonistic relationship between lncRNAs and miRNAs can have profound effects on gene regulatory networks and cellular phenotypes.

Overall, the antagonistic interactions between lncRNAs and miRNAs are dynamic and multifaceted, contributing to the complexity of gene regulation. These interactions allow for fine-tuning of gene expression, governing cellular processes, and impacting various biological contexts, including development, disease, and response to therapy. Antagonistic interactions between long non-coding RNAs (lncRNAs) and microRNAs (miRNAs) have the potential to promote the exaggerated activation of tumor progression pathways or inhibit “self” regulatory responses in non-tumor cells within the cellular microenvironment.

In certain cases, lncRNAs can act as molecular sponges or decoys for specific miRNAs, sequestering them and preventing them from binding to their target mRNAs. This leads to the upregulation of genes that are normally targeted by the miRNAs. These genes can include oncogenes or factors involved in tumor progression pathways, thereby promoting tumor growth and metastasis (Salmena et al., 2011).

Additionally, lncRNA-miRNA interactions can impact non-tumor cells within the microenvironment. Immune cells and stromal cells play essential roles in modulating the anti-tumor immune response and maintaining tissue homeostasis. Dysregulation of lncRNAs and miRNAs in these non-tumor cells can disrupt “self” regulatory responses, leading to the suppression of immune surveillance and a supportive microenvironment for tumor growth (Valadi et al., 2007).

These antagonistic interactions between lncRNAs and miRNAs can therefore contribute to the activation of tumor progression pathways and perturb the normal regulatory responses of non-tumor cells within the cellular microenvironment. Understanding the specific lncRNA-miRNA pairs involved and their functional consequences is crucial for comprehending the complex interplay between these molecules and their impact on cancer development and the tumor microenvironment.

## 5 Exosomal circRNAs and lncRNAs in GC

Exosomal long non-coding RNAs (lncRNAs) and microRNAs (miRNAs) have gained considerable attention as potential biomarkers for resistance to therapy and have emerged as promising candidates for developing effective therapeutic strategies in various diseases, including cancer. Exosomes are small extracellular vesicles that are secreted by various cell types and contain a cargo of genetic material, including lncRNAs and miRNAs. These exosomal lncRNAs and miRNAs can be released into the circulation and taken up by recipient cells, where they can remodel the gene expression landscape and influence cellular processes. One of the major advantages of using exosomal lncRNAs and miRNAs as biomarkers for resistance is their stability and presence in various body fluids, such as blood, urine, and saliva. Exosomes protect these cargo molecules from degradation, making them ideal candidates for non-invasive diagnostic purposes. Changes in the levels or expression patterns of specific exosomal lncRNAs and miRNAs have been associated with resistance to therapy, indicating their potential as predictive biomarkers (Chen et al., 2017). Moreover, exosomal lncRNAs and miRNAs have emerged as key players in mediating resistance pathways and can serve as potential therapeutic targets. Experimental evidence suggests that exosomal lncRNAs and miRNAs can modulate cellular responses to therapy by regulating drug efflux, cell survival pathways, and DNA damage repair mechanisms. Targeting these molecules or pathways involved in resistance can lead to the development of more effective therapeutic strategies (Lobb et al., 2015). In addition to being predictive biomarkers and therapeutic targets, exosomal lncRNAs and miRNAs also hold potential as drug delivery vehicles. Exosomes can be engineered to carry specific lncRNAs or miRNAs and delivered to target cells, enabling precise modulation of gene expression and potentially overcoming resistance mechanisms. In summary, exosomal lncRNAs and miRNAs have emerged as promising biomarkers for resistance to therapy and hold substantial potential for developing effective therapeutic strategies. Further research and validation are needed to identify specific exosomal lncRNA and miRNA signatures associated with resistance, understand their functional roles in resistance mechanisms, and explore their therapeutic applications in precision medicine.

### 5.1 The role of exosomal circRNAs in the development of GC

A group of exosomal circular RNAs (circRNAs) exhibit abnormal expression in gastric cancer (GC) cells and tissues, which may significantly contribute to the occurrence, development, and progression of GC. CircRNAs are resistant to degradation by exonucleases and ribonucleases, which endows them with higher stability and longer half-lives in body fluids. Additionally, the expression of circRNAs in exosomes is highly sensitive and specific in different stages of GC, making them attractive biomarkers for GC diagnosis and prognosis (Lu L. et al., 2021). The potential roles and mechanisms of exosomal circRNAs in GC are listed in Table 1. For instance, studies have found that cirs-133 regulates browning of white adipose tissue in GC patients, and its inhibition markedly reduces tumor-bearing mice’s oxygen consumption and heat production capacity, consequently restraining tumor growth (Zhang H. et al., 2019). Furthermore, the expression of circNRIP1 is significantly elevated in GC patient tissues, indicating poor prognosis, and its inhibition suppressed the proliferative, migratory, and invasive abilities of GC cells. CircNRIP1 also promotes intercellular communication in GC cells by sponging miR-149-5p and increasing AKT1 expression, thus facilitating the malignant progression of GC (Zhang et al., 2019c). In particular, Circ-RANGAP1 expression in plasma exosomes of GC patients increases significantly, suggesting a negative prognostic influence. Further studies indicate that circ-rangap1 overexpression promotes GC cell migration and invasion, as well as tumor growth and metastasis, by transferring to GC cells through the miR-877-3p/VEGFA axis (Lu et al., 2020). The expression of CircSHKBP1 in GC tissues is also related to TNM staging and poor prognosis, and its overexpression significantly enhances GC cell growth, metastasis, and angiogenesis. Mechanisms experiments suggest that circSHKBP1 enhances the stability of VEGF mRNA by binding to miR-582-3p, increasing HUR expression, and blocking STUB1-HSP90 interaction. Moreover, circSHKBP1 directly binds to HSP90, thereby inhibiting the ubiquitination of HSP90 and promoting the malignant progression of GC (Xie et al., 2020). Similarly, circnhsl1 promotes GC cell migration, invasion, and glutamine catabolism by sponging miR-149-5p and promoting YWHAZ expression (Hui et al., 2020). Furthermore, inhibition of Circ_0000260 significantly suppresses the chemical resistance of CDDP, cell growth, and metastasis, and promotes apoptosis, possibly by decreasing the expression of MMP11 via miR-129-5p binding (Liu et al., 2020). In addition, exosomal circ_0032821, circNEK9, Circ29, and circUBE2Q2 promote chemoresistance, migration, invasion, and proliferation of OXA-sensitive GC cells, promoting GC occurrence and progression via diverse signaling pathways and mechanisms (Yang J. et al., 2021; Li S. et al., 2021; Zhong et al., 2021). Circrell1 can inhibit GC cell proliferation, migration, invasion, and anti-apoptosis, partly by binding to miR-637 and indirectly enhancing EPHB3 expression through autophagic activation (Sang et al., 2022). Finally, CircITCH inhibits GC metastasis by sponging miR-199a-5p and increasing Klotho expression, which is a potential biomarker for GC occurrence and development (Wang et al., 2021c). In conclusion, due to their specific tissue, developmental stage and tumor type conservation, circRNAs play a significant role in GC. Exosomes protect circRNA degradation and facilitate the intercellular transmission of GC-related circRNAs, making exosomal circRNA a potential valuable for GC diagnosis and prognosis.

**TABLE 1:** Potential role and mechanism of exosomal circRNAs and lncRNAs in GC.

CircRNAs	Parent cell/source	Target cell	Mechanism	Biological function	References
ciRS-133	GC cells	preadipocytes	miR-133/PRDM16	Promotes differentiation of preadipocytes into brown-like cells	Zhang et al. (2019b)
CircNRIP1	GC cells	GC cells	miR-149-5p/AKT1	Promotes cell proliferation, migration and invasion	Zhang et al. (2019c)
Circ-RanGAP1	Plasma	GC cells	miR-877–3p/VEGFA	Promotes cell proliferation, migration and invasion	Lu et al. (2020)
CircSHKBP1	Plasma	GC cells	miR-582-3p/HUR/VEGF	Promotes cell proliferation, migration and invasion	Xie et al. (2020)
CircNHSL1	Plasma	GC cells	miR-149-5p/YWHAZ	Promotes cel migration and invasion and breakdown of glutamine	Hui et al. (2020)
circ_0000260	CDDP resistant GC cells	CDDP sensitive GC cells	miR-129-5p/MMP11	Promote CDDP chemoresistant	Liu et al. (2020)
circ_0032821	OXA-resistant GC cells	OXA-sensitive GC cells	miR-515-5p/SOX9	Enhance OXA resistance	Zhong et al. (2021)
circNEK9	GC cells	GC cells	miR-409-3p/MAP7	Promotes cell proliferation, migration and invasion	Yu et al. (2021b)
circ_0044366	GC cells	HUVECs	miR-29a/VEGF	Inhibits cell proliferation, migration, tube formation	Li et al. (2021d)
circ-UBE2Q2	GC cells	GC cells	miR-370-3p-STAT3	Promotes tumorigenicity and metastatic potential of GC	Yang et al. (2021b)
CircRELL1	GC cells	GC cells	miR-637/EPHB3	Promotes cell proliferation, migration and invasion	Sang et al. (2022)
LncRNAs					
LncHOTTIP	Cisplatin-resistant GC cells	Cisplatin-sensitive GC cells	miR-218/HMGA1	Promotes resistance to cisplatin	Wang et al. (2019b)
LncPCGEM1	HGCs	NGCs	SNAI1	Promotes cell migration and invasion	Piao et al. (2021)
LINC01559	MSCs	GC cells	miR-1343-3p/PGK1/PI3K/AKT	Promotes cell proliferation, migration and stemness	Wang et al. (2020)
FRLnc1	GC cells	GC cells	—	Promotes cell proliferation, migration and invasion	Zhang et al. (2021c)
LncHOXA10	Plasma	GES-1	ATRA/LncHOXA10/PC	Promotes GC malignant progression	Wang et al. (2022d)
LncCRNDE	M2 polarized macrophages	GC cells	NEDD4-1/PTEN	Promotes the proliferative ability and the resistance to CDDP	Xin et al. (2021a)
lncRNA HCG18	GC cells	M2 polarized macrophages	miR-875-3p/KLF4	Promotes M2 macrophage polarization	Xin et al. (2021b)
SND1-IT1	GC cells	GES-1	miR-1245b-5p/DDX54/USP3	Promotes GC malignant progression	Jin et al. (2022)

### 5.2 Role of exosomal circRNAs in the diagnosis and prognosis assessment of GC

Several studies have investigated the potential of circRNA in the exocrine body to serve as a biomarker for diagnosing GC. The significant heterogeneity of exosomal circRNAs offers promising prospects for the diagnosis, staging, and treatment of tumors. Table 2 displays evidence of the potential of exosomal circRNAs as diagnostic and prognostic tools in GC. For instance, Shao et al. studied exosomes from 41 healthy volunteers and 39 EGC patients, measuring levels of hsa_circ_0065149 through qrt-PCR, and found that the levels of hsa_circ_0065149 in plasma exosomes of EGC patients were significantly lower than those in healthy controls. ROC curves demonstrated an AUC of 0.640 (95% CI, 0.509–0.771; *p* = 0.031) for hsa_circ_0065149 in plasma exosomes as a good early diagnostic marker for EGC (Shao et al., 2020). In another study, Zheng et al. identified specific expression of circRNA in GC secretions by circRNA microarray, and they found 58 circRNAs that were considerably upregulated in secretions from GC cells. They validated these results with samples from 60 patients with primary gastric cancer, 30 patients with chronic gastritis, and 30 healthy subjects. The expression of exosomal hsa_circ_0015286 was significantly higher in GC tissues, plasma, and cancer cells. Moreover, those with high expression of hsa_circ_0015286 had shorter overall survival (OS) than those with low expression. ROC curve analysis verified that the AUC of hsa_circ_0015286 was 0.778. In addition, they found that the expression of exosomal hsa_circ_0015286 was strongly correlated with tumor size, TNM staging, and lymph node metastasis (Zheng et al., 2022). Wang et al. determined that the expression level of circ-ITCH in serum-derived secretions from GC patients was considerably lower than that in serum-derived secretions from healthy donors. By constructing an ROC curve, they found that its AUC value was 65.38% lower, leading to diagnostic applications in GC (Wang et al., 2021c).

**TABLE 2:** Potential of exosomal circRNAs as dianostic and prognostic tools in GC.

Exosomal circRNA	Sample size	Detection Method	*p*-value	Diagnosis	TNM	LNM	DM	OS	DFS	Follow-up	References
	(Normal:Tumor)				(*p*-value)	(*p*-value)	(*p*-value)	(*p*-value)	(*p*-value)	(months)	
Hsa_circ_0065149	(41 : 39)	Specific	*p* < 0.001	AUC = 0.640	—	—	—	—	—	—	Shao et al. (2020)
		qRT-PCR									
Hsa_circ_0015286	(30 : 60)	Specific	*p* < 0.001	AUC = 0.778	*p* < 0.001	*p* < 0.001		*p* < 0.01		40	Zheng et al. (2022)
		qRT-PCR									
Circ-ITCH	(33 : 33)	Specific	*p* < 0.05	AUC = 0.654	—	—	—	—	—	—	Wang et al. (2021c)
		qRT-PCR									

### 5.3 The role of exosomal lncRNAs in the development of GC

Exosomal transmission of lncRNAs has been found to contribute to the progression of various cancers, including gastric cancer (GC) (Xin et al., 2021a). Table 1 summarizes the potential roles and mechanisms of exosomal lncRNAs in GC. Specifically, the upregulation of LncHOTTIP expression in cisplatin-resistant GC cells has been shown to promote cisplatin resistance and inhibit sensitivity to the drug. Moreover, the exosome-mediated transmission of HOTTIP from drug-resistant to drug-sensitive cells further promotes cisplatin resistance through the regulation of the miR-218/HMGA1 axis (Wang J. et al., 2019). Another lncRNA, Lncpcgem 1, can promote cisplatin resistance and cell invasion/migration in GC by stabilizing SNAI1, which in turn induces epithelial mesenchymal transformation (EMT) (Piao et al., 2021). Similarly, the exosome-mediated transmission of LINC01559 from mesenchymal stem cells (MSCs) to GC cells promotes cell proliferation, migration, and dryness in GC by upregulating PGK1 expression and inducing PTEN promoter methylation (Wang et al., 2020). Furthermore, FRLNC1 expression is elevated in GC tumors, serum, and exosomes, which can promote GC growth and metastasis via its transfer to GC cells (Zhang Y. et al., 2021). Exosomal LncHOXA10 is also markedly elevated in serum and gastric tissues of patients with gastric precancerous lesions (GPL), promoting the malignant progression of GES-1 by interacting with pyruvate carboxylase (PC). All-trans retinoic acid (ATRA) can attenuate the progression and malignancy of GPL by reducing the expression of LncHOXA10 and PC in exosomes (Wang C. et al., 2022). Another lncRNA, CRNDE, is enriched in M2-polarized macrophage-derived exosomes and promotes CDDP resistance and proliferation capacity of GC cells by promoting NEDD4-1-mediated ubiquitination of PTEN (Xin et al., 2021a). GCCS-Exos are shown to promote M2 macrophage polarization by delivering HCG18, which regulates KLF4 expression (Xin et al., 2021b). Lastly, SND1-IT1 is significantly overexpressed in GC cell-secreted exosomes and promotes malignant transformation of GES-1 by increasing USP3 expression and mediating deubiquitination of SNAIL1 (Jin et al., 2022).

### 5.4 The role of exosomal lncRNAs in the diagnosis and prognosis assessment of GC

Human blood contains a substantial amount of long non-coding RNAs (lncRNAs) besides microRNAs, which can be detected through RNA sequencing technology or microarray analysis and confirmed via RT-PCR. This shows the promise of circulating lncRNAs in the detection of gastric cancer (GC) (Huang and Yu, 2015). Lin et al. conducted high-throughput sequencing screening and sample analysis and discovered that the expression of lncUEGC1 and lncUEGC2 was significantly higher in the secretions from GC cells and early GC (EGC) patients. Further experiments showed that lncUEGC1 was stably encapsulated into the plasma secretions from GC patients and effectively distinguished EGC patients from healthy individuals and precancerous chronic atrophic gastritis patients (Lin et al., 2018). Li et al. isolated serum exosomes from GC patients and healthy subjects and found that the expression level of exosomal lnc-GNAQ-6:1 in the serum of GC patients was significantly higher than that in healthy volunteers. Additionally, the AUC value of lnc-GNAQ-6:1 in diagnosing GC patients was 0.732 (Li et al., 2020). Cai et al. discovered that lncPCSK2-2:1 was significantly downregulated in the serum secretions of GC patients compared to healthy individuals, and its expression level was negatively correlated with tumor size, tumor stage, and venous infiltration. Furthermore, lncPCSK-2:1 was highly accurate in diagnosing GC, with an AUC of 0.896 (Cai et al., 2019). Increased expression of CEBPA-AS1 was found in both GC cells and exosomes secreted by GC cells, and most CEBPA-AS1 could be encapsulated in plasma exosomes. The AUC value of CEBPA-AS1 in distinguishing GC patients from healthy controls was 0.824 (Piao et al., 2020). Xu et al. reported that the expression level of serum MIAT in GC patients was significantly higher than that in healthy controls and positively correlated with poor prognosis and survival rates (Xu et al., 2020). The expression level of H19 in the serum exocrine body of GC patients was significantly increased before and after surgery and significantly decreased after surgery. Moreover, the level of H19 was significantly correlated with the TNM stage, and the ROC AUC value was 0.849, indicating the diagnostic and prognostic value of H19 in GC (Zhou H. et al., 2020). Zhang et al. revealed that FRLNC1 expression was significantly upregulated in serum exosomes of GC patients, and high expression of FRLNC1 was strongly correlated with lymph node metastasis (LNM) and TNM staging with an ROC AUC value of 0.863 (Zhang Y. et al., 2021). Song et al. investigated the expression level of exosomal lncgc1 and its prognostic correlation with GC patients, finding that exosomal lncgc1 was more accurate in predicting disease-free and overall survival than a prognostic risk stratification model for AJCC staging (Song et al., 2022b). The evidence supports the potential use of exosomal lncRNAs in the diagnosis and prognosis of GC (Table 3).

**TABLE 3:** Potential of exosomal lncRNAs as dianostic and prognostic tools in GC.

Exosomal circRNA	Sample size	Detection Method	*p*-value	Diagnosis	TNM	LNM	DM	OS	RFS	Follow-up	References
	(Normal:Tumor)				(*p*-value)	(*p*-value)	(*p*-value)	(*p*-value)	(*p*-value)	(months)	
lncUEGC1	(60 : 69)	Specific	*p* < 0.001	AUC = 0.876	—	—	—	—	—	—	Lin et al. (2018)
		qRT-PCR									
lnc-GNAQ-6:1	(43 : 27)	Specific	*p* < 0.001	AUC = 0.732	—	—	—	—	—	—	Li et al. (2020)
		qRT-PCR									
lncPCSK2-2:1	(29 : 63)	Specific	*p* = 0.006	AUC = 0.896	*p* = 0.006	*p* = 0.488	—	—	—	—	Cai et al. (2019)
		qRT-PCR									
CEBPA-AS1	(40 : 40)	Specific	*p* < 0.01	AUC = 0.826	*p* < 0.001	—	—	—	—	—	Piao et al. (2020)
		qRT-PCR									
MIAT	(50 : 109)	Specific	*p* < 0.001	—	*p* < 0.001	*p* = 0.0006	*p* = 0.096	*p* = 0.02	*p* = 0.002	60	Xu et al. (2020)
		qRT-PCR									
H19	(81 : 81)	Specific	*p* < 0.05	AUC = 0.849		*p* = 0.007	*p* = 0.271	—	—	—	Zhou et al. (2020b)
		qRT-PCR									
FRLnc1	(60 : 60)	Specific	*p* < 0.01	AUC = 0.863	*p* = 0.009	*p* = 0.004	—	—	—	—	Zhang et al. (2021c)

### 5.5 Immune component of interactions and its relevance in the diagnosis and treatment of gastric cancer

The immune component of interactions plays a crucial role in the diagnosis and treatment of gastric cancer. The immune system has the ability to recognize and eliminate cancer cells, and its dysregulation is often associated with tumor development and progression. In recent years, immunotherapy has emerged as a promising approach for the treatment of gastric cancer by enhancing the body’s immune response against tumors.

Exosomal long non-coding RNAs (lncRNAs) and microRNAs (miRNAs) have shown potential as biomarkers for immune response in gastric cancer. Exosomes derived from tumor cells can carry specific lncRNAs and miRNAs that regulate immune responses. These exosomal lncRNAs and miRNAs can modulate immune cell functions, such as T cells, natural killer cells, and dendritic cells, by affecting their activation, differentiation, or immunosuppressive properties.

The profiling of exosomal lncRNAs and miRNAs in gastric cancer patients has revealed associations with immune-related processes and patient outcomes. For example, specific lncRNA and miRNA signatures in exosomes have been associated with immune cell infiltration, immune checkpoint molecule expression, and response to immunotherapy in gastric cancer (Xu et al., 2020). Utilizing exosomal lncRNAs and miRNAs as biomarkers for immune response can aid in the diagnosis, prognosis, and prediction of therapeutic response in gastric cancer patients. By monitoring the expression levels or signatures of immune-related exosomal molecules, clinicians can gain insights into the patient’s immune status and potentially identify those who would benefit from immunotherapeutic approaches. Furthermore, exosomal lncRNAs and miRNAs have the potential to guide the development of effective immunotherapeutic strategies. Understanding the roles of specific exosomal molecules in modulating immune responses can help identify targets for immunotherapy interventions. Manipulating the expression or function of exosomal lncRNAs and miRNAs can potentially enhance anti-tumor immune responses or overcome immunosuppressive mechanisms.

Overall, the immune component of interactions in gastric cancer is critical for diagnosis and treatment. Exosomal lncRNAs and miRNAs can serve as valuable biomarkers for immune response and provide insights into the development of effective immunotherapeutic strategies. However, further research is needed to validate the clinical utility of immune-related exosomal molecules and to fully understand their functional roles in the immune landscape of gastric cancer.

## 6 Future expectations and summary

The burgeoning field of RNA research has revealed that both circular RNAs (circRNAs) and long non-coding RNAs (lncRNAs) play critical roles in various physiological and pathological processes. Although extensive studies have been conducted on these RNAs, their formation and clearance mechanisms remain poorly understood. As subcellular vesicles with dimensions in the nanometer range, exosomes bear unique protein and RNA signatures that enable intercellular information exchange and facilitate intercellular communication. Enabled by their high specificity and distinctive features, exosomes that incorporate circRNAs and lncRNAs hold great potential as early diagnostic and prognostic markers for cancer, as well as biomarkers for numerous other diseases. Biomarkers refer to biomolecules that occur in blood, tissues, and bodily fluids and that can be utilized to evaluate the normal and pathological states objectively (Presti et al., 2022; Vandendriessche et al., 2022; Xu et al., 2022). The clinical significance of biomarkers lies in their application for the early detection and diagnosis of various diseases, as well as for monitoring the therapeutic response (Yu I. S. et al., 2022; Davenport et al., 2022; Holper et al., 2022). Due to their stability, sensitivity, and specificity, these typical characteristics of biomarkers allow their use in clinical practice. Precision medicine, particularly liquid biopsy, focuses on identifying cancer biomarkers, which possess exceptional capabilities for detecting indications of cancer risk, guiding patients to receive the most appropriate treatment, and helping clinicians track disease progression or recurrence. Research has identified exosomes as promising cancer biomarkers for diagnostic prognosis and prediction, as they can markedly reduce the complexity of samples compared to systemic body fluids, and require minimally invasive procedures, or even no invasive procedures at all, in the context of liquid biopsies (Rojas et al., 2022; Zhang et al., 2022). Furthermore, given the good biodistribution and biocompatibility of exosomes, it is highly feasible to promote the development of advanced cancer therapies, especially for precision medicine applications.

CircRNAs and lncRNAs have significant implications in the pathogenesis of gastric cancer (GC), whereby disrupted expression of these non-coding RNAs is closely associated with the development and progression of the disease. By influencing key cancer-related signaling pathways, aberrantly expressed circRNAs and lncRNAs can either promote or suppress the progression of GC. Exosomes are potential reservoirs of circRNAs and lncRNAs, which can persist stably in body fluids for prolonged periods of time. Besides reflecting the status of disease progression, treatment response, and regression with timeliness and accuracy, the changes in exosomal circRNA and lncRNA expression hold promise in early diagnosis and prognosis assessment of GC. Moreover, circRNA and lncRNA can serve as prospective therapeutic targets for the development of novel anti-cancer drugs. Therefore, studies have been conducted to utilize exosomes as proficient delivery vehicles for RNA-based therapeutic strategies (Fu et al., 2019). Multiple approaches have been explored, ranging from the transfection of anti-miR-214 to reverse cisplatin resistance, to the use of exosomes embodying HGF siRNA to inhibit the proliferation and migration of GC cells, and to transferring miR-21 inhibitors through macrophage-derived exosomes to curb cell migration and induce apoptosis. Encouragingly, the results suggest that exosomal circRNAs and lncRNAs hold potential as promising therapeutic targets for GC (Wang et al., 2018).

However, challenges and difficulties remain. Exocytosis of exosomal vesicles increases circRNAs and lncRNAs clearance, resulting in low abundance of these biomarkers in exosomes. Challenges remain in the collection, storage, and identification of exosomes in addition to the identification of their source and standardization of their expression level. Most current studies have small sample sizes in a single-center study population, leading to biased results, while accurate statistical analysis of data requires large-scale multicenter experimental studies. Regardless, proteomics, high-throughput sequencing, transcriptomics, and bioinformatics analysis continue to help researchers understand the mechanisms of exosomal circRNA and lncRNA in the development of GC and their clinical applications. It is likely that exosomal circRNAs and lncRNAs will become a hot spot for early GC screening, treatment, efficacy prediction, and prognosis assessment in the future.

Exosomal vehicles possess numerous advantages that make them ideal for evaluating biomarkers for early GC detection and assessing appropriate treatment and monitoring of treatment effectiveness. However, further clarifications are required concerning clinical sample accuracy and standardized purification methods, the main functional components of electric vehicles, as well as the most suitable biomarker identification component. Additionally, while RNA has been the focus of EVs in GC studies for the past decade, further elucidation of the basic mechanism/characteristics of EVs biology in GC is required. A deep understanding of EVs will provide better clinical conversion potential for GC.

## Abbreviations

GC, Gastric cancer; TME, tumor microenvironment; circRNAs, Circular RNAs; ncRNAs, noncoding RNAs; lncRNAs, long non coding RNAs; miRNAs, MicroRNAs; EVs, extracellular vesicles; ILVs, intraluminal vesicles; MVBs, multivesicular bodies; ESCRT, endosomal sorting complex required for transport; STAM, signal transduction bridging molecule; ceRNAs, competing endogenous RNAs; mRNAs, messenger RNAs; WAT, white adipose tissue; GAC, gastric adenocarcinoma; OS, overall survival; HGC, hypoxia-cultured GC cells; NGC, normoxic-cultured GC cells; EMT, epithelial-mesenchymal transition; MSCs, mesenchymal stem cells; GPL, gastric precancerous lesions; PC, pyruvate carboxylase; M2-exo, M2-polarized macrophage-derived exosomes.
